# Assessing the Prevalence and Association of Pulp Stones with Cardiovascular Diseases and Diabetes Mellitus in the Saudi Arabian Population—A CBCT Based Study

**DOI:** 10.3390/ijerph17249293

**Published:** 2020-12-11

**Authors:** Kumar Chandan Srivastava, Deepti Shrivastava, Anil Kumar Nagarajappa, Zafar Ali Khan, Ibrahim A. Alzoubi, Mohammed Assayed Mousa, May Hamza, Anju P. David, Khalid Al-Johani, Mohammed Ghazi Sghaireen, Mohammad Khursheed Alam

**Affiliations:** 1 Oral Medicine & Radiology, Department of Oral and Maxillofacial Surgery & Diagnostic Sciences, College of Dentistry, Jouf University, Sakaka 72345, Saudi Arabia; dr.anil.kumar@jodent.org; 2 Periodontics, Department of Preventive Dentistry, College of Dentistry, Jouf University, Sakaka 72345, Saudi Arabia; sdeepti20@gmail.com (D.S.); dr.ibrahim.alzoubi@jodent.org (I.A.A.); 3 Department of Oral and Maxillofacial Surgery & Diagnostic Sciences, Division of Oral Surgery, College of Dentistry, Jouf University, Sakaka 72345, Saudi Arabia; dr.zafar.khan@jodent.org; 4 Prosthodontics, Prosthetic Dental Sciences, College of Dentistry, Jouf University, Sakaka 72345, Saudi Arabia; dr.mohammed.mousa@jodent.org (M.A.M.); dr.may.hamza@jodent.org (M.H.); dr.mohammed.sghaireen@jodent.org (M.G.S.); 5 Research Fellow, Prosthodontic Unit, School of Dental Sciences, Health Campus, Universiti Sains Malaysia, Kubang Kerian, Kota bharu 16150, Kelantan, Malaysia; 6 Registrar at Al-Jouf Specialist Dental Centre, Ministry of Health, Sakaka 72345, Saudi Arabia; anjupdavid@gmail.com; 7 Department of Oral Diagnostic Sciences, Faculty of Dentistry, King Abdulaziz University, Jeddah 80200, Saudi Arabia; kauoralmed@gmail.com; 8 Orthodontics, Department of Preventive Dentistry, College of Dentistry, Jouf University, Sakakah 72345, Saudi Arabia; dralam@gmail.com

**Keywords:** oral health, three-dimensional imaging, cone beam CT, pulp stone, cardiovascular disease, diabetes mellitus, systemic diseases

## Abstract

The detection of pulp stone in a patient suffering from undiagnosed systemic diseases can be an early diagnostic indicator. Thus, the aim of the study was to assess the prevalence of pulp stones in the Saudi Arabian population with cardiovascular diseases and diabetes mellitus. In a retrospective study, we included cone-beam computed tomography (CBCT) scans of 73 patients with cardiovascular disease and 76 patients with diabetes mellitus as group I and II, respectively. Group III comprised of CBCT scan of 80 healthy controls. From a total of 229 scans, 4807 teeth were screened for pulp stones throughout the arches. A chi-square test was used for comparing the prevalence of pulp stones among the groups. Univariable and multivariable analysis was done to evaluate the independent risk indicators for pulp stones. The tooth-wise prevalence of pulp stones in group I, II, and III was found to be 16.65%, 9.01%, and 3.86%, respectively. Patient-wise (*p* < 0.01) and tooth-wise (*p* < 0.01) prevalence was recorded significantly highest in the cardiovascular group followed by the diabetic group. The control group had the least prevalence. Significantly (*p* < 0.01) higher number of pulp stones were found in cardiovascular patients with age > 50 years compared to other groups. Similarly, a significantly increased number of pulp stones were seen in the 1st molar (*p* < 0.05) and the maxillary jaw (*p* < 0.05) of patients with cardiovascular diseases. Subjects with cardiovascular disease and diabetes were found to have 2.94 times (*p* < 0.001; CI 1.54–3.10) and 1.81 times (*p* < 0.01; CI 0.48–2.06) higher risk of having pulp stones in comparison to healthy subjects. The first molar has 2.20 times (*p* < 0.001; CI 0.84–2.45) increased the risk of having pulp stones compared to other tooth types. Systemic disease such as cardiovascular disease and diabetes mellitus poses a higher risk for the development of pulp stones. Among the systemic disease group, patients in the cardiovascular group showed a higher risk for pulp stones and also reported the maximum number of pulp stones compared to the diabetic and healthy subjects.

## 1. Introduction

Pulp stones (PS) are compact degenerative masses of calcified tissues located in the dental pulp [1]. These PS may be found in coronal or radicular portion of the pulp. However, coronals PS are more prevalent compared to the radicular [1]. PS varies in size and number, where some might be very small in size and other may be large to occlude the pulp chamber. These PS may be free, embedded or adherent to the wall of pulp space [2]. True PS are formed by dentine and lined by odontoblast, whereas, false PS are formed from degenerative cells which gets mineralized [2]. They can be found in both primary and permanent dentition [1].

Normally the PS are asymptomatic unless they encroach any nerve fiber bundles [2]. Idiopathic pain is sometimes associated with PS and pulp calcification (PC). The adherent and embedded PS poses much difficulty during root canal treatment. They cause severe occlusion in the endodontic treatment if present along the root curvature [2]. Larger PS can cause significant blockage of the canal and changes the internal anatomy.

There is no confirmatory etiopathogenesis of PS formation. However, various factors are associated with its formation such as increasing age, prolonged stimulation from caries and deep restorations, orthodontic treatment, periodontal disease, genetics, and systemic diseases [1,3,4]. Within the oral cavity, the dental plaque plays an important role in periodontal health and disease. It is a well-established fact that periodontal disease can contribute to systemic illness [5,6]. Conversely, the presence and severity of the systemic conditions have shown to adversely impact the oral health [7,8].

Recently, PS has been associated with many systemic conditions namely coronary artery disease (CAD), diabetes mellitus (DM), renal diseases and autoimmune diseases. Few authors have reported a significant association between renal calculi and PS [9]. Moreover, researchers believe that PS detection can be a diagnostic marker of systemic disease [10,11]. Cardiovascular disease (CVD) is a major concern of morbidity and mortality in the modern era. Atherosclerosis is the major causative factor for CAD leading to ischemic heart disease. It has been found by many authors that CVD can be associated with PS [12,13]. Recently, hypertension (HTN) has also been linked with PS [14]. The above-mentioned systemic conditions are also known to alter the harmonious balance among the oral microbial flora, leading to an upsurge in the virulent types of bacteria. This condition is referred to as oral dysbiosis, which in turn converts the environment conducive for inflammatory events. Studies have traced raised levels of pro-inflammatory cytokines to support this phenomenon. The dysbiosis predispose not only the periodontal diseases, but also propels the underlying systemic conditions [15,16]. Additionally, the adverse habit has also shown to contribute in the progression of periodontal diseases by altering the microbiota and inflammatory responses [17].

Researchers have found PS with various radiographic techniques including intraoral periapical radiograph (IOPA), bitewing and orthopantomography (OPG) [4,18]. In the modern era, cone beam computed tomography (CBCT) is quite often used for complex endodontic procedures; prosthodontics-implant and morphometric analysis of anatomical structures [19,20,21,22]. The added advantages of CBCT are being three-dimensional, giving a holistic image in all the sections. Hence CBCT gives additional information to arrive a diagnosis and formulation of treatment plan [23].

In Saudi Arabia, the relationship of PS with systemic disease has not been studied extensively. Thus, considering the above observation, our study aimed at evaluating the prevalence pulp stone in the Saudi Arabian population suffering from cardiovascular diseases and diabetes mellitus.

## 2. Materials and Methods

*Study identification:* A retrospective, observational study in a hospital setting (faculty of dentistry, Saudi Arabia) was conducted. Prior to study, we got ethical approval from the institutional ethics committee (EAC/203/16).

*Sample identification:* We retrieved 256 patients’ CBCT scans that underwent scanning for diagnosis and treatment needs during the time span of 2 years (2016–2018). Twenty-seven (27) scans with issues such as incomplete coverage of maxillary and mandibular arches, poor quality, completely edentulous arches, and pediatric patient were excluded. We included 229 scans and based on the patient’s systemic condition, categorized them into three study groups. The first group (I) included 73 patients with a chronic history of CVD (including ischemic heart disease, HTN). The second group (II) included 76 patients whose medical history revealed DM. The control group composed of 80 healthy subjects with no history of any systemic disease. We excluded patients with a history of renal disease or gout from the study. Based on age, the patients were categorized as younger (20–35), middle (35–50) and elderly age (>50 years). From 229 patients, we examined 4807 teeth, including both arches. The exclusion criteria also included teeth with deep caries, restoration, or periapical infection.

**Figure 1:**
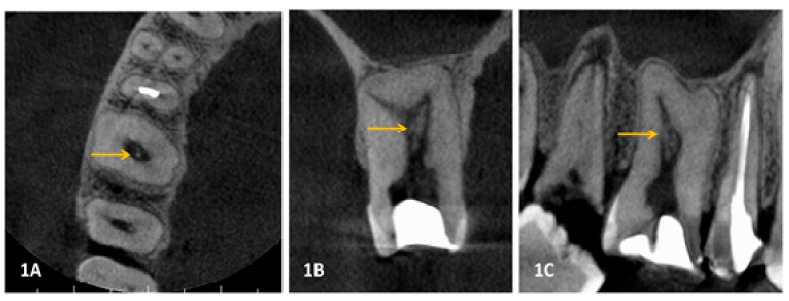
This is a figure depicting the CBCT scan images in three different planes showing (orange arrow) radiopaque mass in the root canal of right maxillary 1st molar right maxillary arch (1**A**) CBCT axial view; (1**B**) CBCT coronal view; (1**C**) CBCT sagittal view.

*Image analysis:* Patients underwent CBCT scanning with Veraviewepocs 3D (J. Morita, 7129 Komuro, Ina, Kitaadachi District, Saitama 362-0806, Japan). The exposure setting used while scanning included 60–70 kV, 10 mA and 40 s of exposure. The machine had Voxel size of 0.125 mm with a different field of views. Interpretation of images was done with the OnDemand3D software by two experienced oral radiologists. Both examiners underwent a blinded training session to standardize the interpretation. Inter and intra-examiner reliability was assessed with Cronbach’s correlation coefficient resulted in 0.91 and 0.93, showing acceptable agreement. We screened individual CBCT scan in all planes, namely sagittal, coronal, and axial in both the arches for a discrete radiopaque mass in the coronal and radicular pulp (Figure 1).

*Statistical analysis:* The data was collected and transferred into a Microsoft Excel sheet. Later, sample characteristics along with the prevalence of PS were presented in number and percentages. We used Chi-square test at 95% confidence interval for inferential analysis on SPSS version 21. Univariable and multivariable logistic regression analysis was carried out to assess the odds ratio and adjusted odds ratio respectively for the independent risk factors. The *p* value at <0.05 was considered significant.

## 3. Results

The study investigated the prevalence of the PS in three study groups. In each group, majority of the participants were above 50 years, with mean age of 56.4 ± 8.61, 58.2 ± 5.81, and 55.7 ± 7.42 respectively. Group (I) had 73 subjects with 1585 teeth, whereas group (II) had 76 patients and 1564 teeth included in analysis. Eighty Healthy subjects of group (III) had 1658 teeth examined. The description of the total number of teeth assessed in different segments of jaw and tooth category are explained in Table 1.

According to the primary objective of the study, the overall prevalence of the PS was calculated patient-wise and tooth-wise. Both ways, PS were found to be significantly (*p* < 0.01) most prevalent in patients with cardiovascular disease, followed by diabetic and the least in healthy subjects. Tooth-wise, group I had a prevalence of 16.65%, whereas, group II had 9.01%. Healthy subjects showed the least prevalence (3.86%) among the groups. Subsequently, prevalence pattern with respect to age, gender, arch, and tooth type was observed among the groups. Irrespective of the study group, older-aged (˃50 years) patients have higher prevalence of PS compared to younger and middle-aged patients. However, on an intergroup comparison, significant difference (*p* < 0.01) in the prevalence was observed in ˃50-year age group, with group-I recorded the highest number of PS. Among the type of tooth, 1stmolar recorded the highest number of PS, irrespective of study group. When compared between the groups, patients in group-I significantly (*p* < 0.05) outscored the other groups (Table 2).

On assessing prevalence of PS region-wise, significant difference (*p* < 0.001) was observed with respect to maxillary arch with 19.5%, 11.42%, and 4.13% of PS found in group I, II, and III respectively. Likewise, when tooth-specific prevalence was assessed for 1st molar, 35.02%, 17.52%, and 7.87% of PS was found to be significantly different (*p* < 0.01) in group I, II, and III, respectively (Table 3).

Later, exploration about the independent variables such as the study group, age, gender, jaw, region and tooth type were carried out. Systemic disease has emerged as the promising risk indicator in the development of PS. Among systemic diseases considered in the present study, patients with cardiovascular disease has significantly (*p* < 0.001) 2.94 times higher risk for PS, whereas diabetic patients were shown to have (*p* < 0.01) 1.18 times higher risk compared to the healthy subjects. Age and gender did not pose any significant risk for the PS. Considering the jaw, maxilla has reported 2.09 times (*p* < 0.01) higher risk for PS in contrast to the lower jaw. Among the tooth type, the 1st Molar turned to have 2.20 times (*p* < 0.001) raised risk to have PS (Table 4).

## 4. Discussion

The PS are usually reported as an incidental finding on a radiograph. It can also be found during a pre-operative radiograph or intra-operative root canal treatment [2]. The prevalence of pulp stone reported with various 2D radiographic techniques such as IOPA, bitewing, and OPG, ranges from 8–90%, [1] whereas histological section has revealed greater percentage [4]. Both of these techniques have their own limitations. Two-dimensional radiographic technologies have superimposition of structures and distortion of the images, thus obscuring the visibility of PS. Similarly, in histological section the area of interest might have been skipped while sectioning the tooth. Considering these factors, visualization of PS on CBCT is comparatively easier as it can be visible in various planes provided the PS size should be larger than 200 microns [24]. Nonetheless, the usage of CBCT should be reserved for exploratory purpose after the encouraging initial examination with 2D radiographic technique.

In the present study, the CBCT scans were retrieved from the radiology department for the designated study timeframe. Based on the inclusion and exclusion criteria, we randomly included the scan for the analysis, thus minimized the selection bias in the study.

Nowadays, PS has been considered as an initial risk factor detected at an early phase of the CVD [13]. It is believed that calcification seen in the various regions such as kidney, joints, teeth, and in atherosclerotic plaque are primarily made up of calcium phosphate crystals. These crystals can precipitate an acute immunological reaction leading to an inflammatory response within the arteries. Such event becomes a leading cause for ischemic heart disease causing significant mortality and morbidity [12]. According to other theory, calcifying nanoparticles classified nanobacteria’s are the key factors for the pathological calcifications seen in the gall stones, joint calcification, renal calculi, atherosclerotic plaque and PS [12,25]. In the recent past, osteopontin, a new component of atherosclerotic plaques, was reported to play a role in plaque calcification [13].

In our study the teeth-wise prevalence of pulp stone found in the cardiovascular group patients was 16.65%. It was significantly higher in maxillary arch (10.03%) and in the 1stmolar (4.35%). We also have observed that the cardiovascular disease has emerged as an important risk indicatorfor the development of PS. It’s occurrence in this group was 2.94 times higher compared to healthy subjects. Studies by other researcher have found the most prevalent group with systemic disease having PS was cardiovascular patients [26]. In another study by Edds et al. [27] and Khojostepour et al. [28] 74% of the patients with ischemic heart disease and 68.2% of the subjects with CVS disorder had PS respectively. Another study by Ezoddini-Ardakani et al. found that 67.3% of the individual had PS with ischemic heart disease. Bernick et al. found the narrowing of the lumen of extirpated dental pulp vessels of cardiovascular patients [29]. However, Yilmaz et al. reported hypertension as a non-significant risk factor for the pulp stone [30].

In our study the second most prevalent group where pulp stone was found maximum was DM group. DM is known to compromise the oral and systemic health status of an individual by altering the immune mechanism and eventually compromising the quality of life [31].These subjects were shown to have 1.81 times higher risk for PS compared to the healthy subjects. Similar results were shown in a study by Yilmaz et al. [30]. The probable reason for such observation could be because the dental pulp of patients with DM shows signs of ageing because of obliterative endarteritis. Since the dental pulp has less or no collateral blood circulation in a fully developed tooth, it is more prone for calcification [26].Within the diabetic group the maximum pulp stone was seen in 9.01% and maxillary arch (5.88%) has more pulp stone compared to mandibular arch (3.18%).In a study Nayak et al. [26] they found 7.69 teeth with pulp stone, which is comparable to our study.

The maximum prevalence of PS was found in the maxillary arch and in molars irrespective of all the groups. Moreover, the results showed the maxillary jaw to have 2.09 times higher risk for developing PS compared to the mandibular jaw. Furthermore, the 1st molar appeared to bear the highest risk (2.20 times) for PS. These findings are consistent with other studies [32,33]. The highest prevalence attributed with these teeth could be because of the larger pulp space volume with enormous pulp tissue and better blood supply leading to the precipitation of the calcification [33]. Contrary to our results Satheesh Kumar et al. found more pulp stone in mandible compared to maxilla [10].

Another interesting finding of the study is that the prevalence of pulp stone was greater in age group above 50 years irrespective of the groups. However, it did not appear as an important risk indicator on multivariable analysis. Conflicting results are found in a study where highest prevalence 89.7% was found in 31–40 age group followed by 41–50 years (88.3%) [10]. It is a common belief that as the age advances there is decrease in the number of cells residing in the pulp. Along with this there might be increase in mucopolysaccharides and fibrous components which helps in progression of calcification [32].

It is noteworthy that in the present study any tooth with caries or pathologic wear or restored teeth has been excluded. As these factors can be a confounding factor which might affect the results in any of the groups selected. It is a well-established fact that teeth with carious and abnormal wear has shown more pulp stone formation because of the constant irritation and inflammatory process calcific changes are reported as a defense mechanism of pulp. Hence other study which might have reported higher prevalence have not excluded carious and restored teeth [33,34].

Despite of encouraging results of the current study, the interpretation of the same should be done with caution. Being a comparative study between patients with systemic disease and healthy subjects, there can bechances of overestimating the risk of PS formation. Additionally, the retrospective nature of the present study is not capable of establishing the causal pathways between the PS and systemic diseases. However, the present study has found a significant association between the variables. Thus, based on the results of this study, the systemic diseases, such as cardiovascular diseases and diabetes mellitus can be considered as the risk indicators for PS.

### Limitations and Future Directions

The limitation of the present study is that other systemic disease such as parathyroid and gastrointestinal disorder patients has not been taken. Since a histological section is not taken the actual number be little more as depicted on CBCT. Furthermore, a prospective cohort study should be designed to deduce the direction of the causal pathway between the variables studied in the present study. Additionally, such a study design will be helpful in exploring the association of PS with chronic inflammatory oral conditions, such as various grades of periodontal disease and oral malignancy. Additionally, the impact of medication in various statuses of HTN and DM (well controlled, poorly controlled, or undiagnosed) and its relationship with the occurrence of pulp stones can be evaluated.

## 5. Conclusions

From the present study it can be concluded that the prevalence of pulp stone was higher in patients with cardiovascular diseases and diabetes mellitus. The cardiovascular group outscores the prevalence of pulp stone with diabetic group. The maximum number of PS was found in maxillary arch and 1stmolars irrespective of the groups. The presence of PS on radiographic examination might suggest a possibility of underlying undiagnosed systemic disease which will require proper referral and further management.

## Figures and Tables

**Table 1:** Descriptive characteristics.

Variable		Group I (Cardiovascular) n = 73	Group II (Diabetes Mellitus) n = 76	Group III (Healthy) n = 80	Total n = 229
Age	20–35 Years	7 (9.58)	8 (10.52)	11 (13.75)	26 (11.35)
	36–50 Years	22 (30.13)	27 (35.52)	25 (31.25)	74 (32.31)
	˃50 Years	44 (60.27)	41 (53.94)	44 (55)	129 (56.33)
Gender	Male	39 (53.42)	37 (48.68)	43 (53.75)	119(51.96
	Female	34 (46.57)	39 (51.31)	37 (46.25)	110 (48.03)
Number of Teeth Examined		1585	1564	1658	4807
Jaw	Maxilla	813 (51.29)	805 (51.47)	847 (51.08)	2465 (51.27)
	Mandible	772 (48.70)	759 (48.52)	811 (48.91)	2342 (48.72)
Region	Anterior	667 (42.08)	673 (43.03)	665 (40.10)	2005 (41.71)
	Posterior	918 (57.91)	891 (56.96)	993 (59.89)	2802 (58.28)
Tooth type	Incisor	449 (28.32)	452 (28.90)	412 (24.84)	1313 (27.31)
	Canine	218 (13.75)	221 (14.13)	253 (15.25)	692 (14.39)
	1st Premolar	225 (14.19)	208 (13.29)	245 (15.31)	678 (14.10)
	2nd Premolar	277 (17.47)	288 (18.41)	267 (16.10)	832 (17.30)
	1st Molar	197 (12.42)	194 (12.40)	216 (13.02)	607 (12.62)
	2nd Molar	219 (13.81)	201 (12.85)	265 (15.98)	685 (14.25)

**Table 2:** Overall prevalence and comparative analysis of pulp stones between the study groups.

Variable		Group I (Cardiovascular)	Group II (Diabetes Mellitus)	Group III (Healthy)	Total	*p* Value
Number of patients examined		73	76	80	229	-
Pulp stones (Patient-wise)		24 (32.87)	18 (23.68)	9 (11.25)	51 (22.27)	<0.01 *
Number of Teeth Examined		1585	1564	1658	4807	-
Pulp stones (Teeth-wise)		264 (16.65)	141 (9.01)	64 (3.86)	469 (9.75)	<0.01 *
Age	20–35 Years	41 (2.58)	17 (1.08)	5 (0.30)	63 (1.31)	0.65
	36–50 Years	59 (3.72)	40 (2.55)	18 (1.08)	117 (2.43)	0.72
	˃50 Years	164 (10.34)	84 (5.37)	41 (2.47)	289 (6.01)	<0.01 *
Gender	Male	125 (7.88)	78 (4.98)	28 (1.68)	231 (4.80)	0.45
	Female	139 (8.79)	63 (4.02)	36 (2.17)	238 (4.95)	0.32
Jaw	Maxilla	159 (10.03)	92 (5.88)	35 (2.11)	286 (5.94)	<0.01 *
	Mandible	105 (6.62)	49 (3.13)	29 (1.74)	183 (3.80)	0.08
Region	Anterior	69 (4.35)	38 (2.42)	11 (0.66)	118 (2.45)	0.06
	Posterior	195 (12.46)	103 (6.58)	53 (3.19)	321 (6.67)	<0.05 *
Tooth type	Incisor	42 (2.64)	26 (1.66)	8 (0.48)	76 (1.58)	0.24
	Canine	27 (1.70)	12 (0.76)	3 (0.18)	42 (0.87)	0.45
	1st Premolar	38 (2.39)	16 (1.02)	9 (0.54)	63 (1.31)	0.94
	2nd Premolar	32 (2.01)	20 (1.27)	12 (0.72)	64 (1.33)	0.96
	1st Molar	69 (4.35)	34 (2.17)	17(1.02)	120 (2.49)	<0.05 *
	2nd Molar	56 (3.53)	33 (2.01)	15 (0.90)	104 (2.16)	<0.05 *

Note-* *p* < 0.05.

**Table 3:** Region and tooth specific prevalence and comparative analysis of pulp stones between study groups.

Variable		Group I (Cardiovascular)			Group II (Diabetes Mellitus)			Group III (Healthy)			*p* Value
		Teeth Examined	Pulp Stones		Teeth Examined	Pulp Stones		Teeth Examined	Pulp Stones		
			n	%		n	%		n	%	
Jaw	Maxilla	813	159	19.55	805	92	11.42	847	35	4.13	<0.01 *
	Mandible	772	105	13.60	759	49	6.45	811	29	3.57	0.06
Tooth type	Incisor	449	42	9.35	452	26	5.75	412	8	1.94	0.24
	Canine	218	27	12.38	221	12	5.42	253	3	1.18	0.45
	1st Premolar	225	38	16.88	208	16	7.69	245	9	3.67	0.94
	2nd Premolar	277	32	11.55	288	20	6.94	267	12	4.94	0.96
	1st Molar	197	69	35.02	194	34	17.52	216	17	7.87	<0.001 *
	2nd Molar	219	56	25.57	201	33	16.41	265	15	5.66	<0.01 *

* *p* < 0.05.

**Table 4:** Univariable and multivariableanalysisassessing the risk factors for the development of Pulp stones.

Parameter		Univariable Analysis		Multivariable Analysis	
		Odds Ratio (CI)	*p* Value	Adjusted Odds Ratio (CI)	*p* Value
Study Group	Cardiovascular	3.44 (1.92–4.10)	<0.01 *	2.94 (1.54–3.10)	<0.001 *
	Diabetes Mellitus	2.18 (0.94–3.76)	<0.01 *	1.81 (0.48–2.06)	<0.01 *
	Control	Reference	-	Reference	-
Age	20–35 Years	Reference	-	-	-
	36–50 Years	1.32 (0.21–1.85)	0.11	-	-
	˃50 Years	1.91 (0.54–2.20)	0.07	-	-
Gender	Male	0.82 (0.14–1.25)	0.74	-	-
	Female	Reference	-	-	-
Region	Anterior	Reference	-	-	-
	Posterior	1.8 (0.34–2.11)	0.24	-	-
Jaw	Maxilla	2.4 (0.72–3.10)	<0.01 *	2.09 (0.45–2.56)	<0.01 *
	Mandible	Reference	-	Reference	-
Tooth type	Incisor	Reference	-	Reference	-
	Canine	1.09 (0.21–1.40)	0.56	1.00 (0.15–1.28)	0.59
	1st Premolar	1.21 (0.38–1.62)	0.62	1.12 (0.22–1.54)	0.44
	2nd Premolar	1.84 (0.48–2.20)	0.91	1.30 (0.38–1.75)	0.25
	1st Molar	2.67 (0.98–3.15)	<0.01 *	2.20 (0.84–2.45)	<0.001 *
	2nd Molar	2.01 (0.65–2.78)	<0.01 *	1.99 (0.45–2.10)	0.08

* Statistically significant.

## Abbreviations

CAD	Coronary artery disease
CBCT	Cone beam computed tomography
CVD	Cardio vascular disease
DM	Diabetes mellitus
HTN	Hypertension
IOPA	Intraoral periapical radiograph (IOPA)
OPG	orthopantomography
PC	pulp calcification
PS	Pulp stone
